# Increasing the Q-factor of Fabry–Perot etalons using focused Bessel beam illumination

**DOI:** 10.1364/OL.505390

**Published:** 2023-12-01

**Authors:** Dylan M. Marques, Oliver Sheppard, James A. Guggenheim, Peter R. T. Munro

**Affiliations:** 1 Institute of Cardiovascular Sciences, University of Birmingham, Birmingham, UK; 2 Department of Medical Physics and Biomedical Engineering, University College London, London, UK; 3 School of Engineering, University of Birmingham, Birmingham, UK

## Abstract

Sensing and filtering applications often require Fabry–Perot (FP) etalons with an Interferometer Transfer Function (ITF) having high visibility, narrow Full Width at Half Maximum (FWHM), and high sensitivity. For the ITF to have these characteristics, the illumination beam must be matched to the modes of the FP cavity. This is challenging when a small illumination element size is needed, as typical focused beams are not matched to the FP cavity modes. Bessel beams are a potential alternative as their structure resembles the FP cavity modes while possessing a focused core. To study the feasibility of using Bessel beam illumination, in this Letter, ITFs of an FP etalon were measured using Bessel and Gaussian illumination beams. A Bessel beam with core size of 28 µm provided an ITF with visibility 3.0 times higher, a FWHM 0.3 times narrower, and a sensitivity 2.2 times higher than a Gaussian beam with waist 32 µm. The results show that Bessel beam illumination can provide ITFs similar to that of collimated beam illumination while also having with a focused core.

## Introduction.

An FP etalon is an interferometer with an optical cavity formed between two parallel mirrors [1]. FP etalons are widely used for filtering [2] and sensing [3] due to their characteristic transfer function. In particular, if designed with a high Q-factor (i.e., having high mirror reflectivities), an FP etalon acts as a mirror throughout most of the spectral range, aside from particular wavelengths at which the FP etalon acts as a nearly transparent device. This particular response of an FP etalon can be seen by measuring the wavelength resolved reflectivity or transmissivity, typically called the ITF. The rapid change exhibited by the ITF at particular wavelengths is due to light resonating inside the FP cavity. In particular, at most wavelengths, light inside the FP cavity interferes destructively with itself causing the FP etalon to act as a mirror. However, if the cavity optical path length matches an integer number of wavelengths, light undergoing different numbers of roundtrips inside the FP cavity interferes constructively, leading to the FP etalon becoming transparent to light.

In general, it is desirable to maximize the contrast between an ITF’s in and out of resonance reflectivity/transmissivity (typically called visibility) and sensitivity (proportional to the ITF derivative) while minimizing the FWHM. These ITF characteristics are achieved when the illumination beam is matched to the FP cavity modes. A mode of the cavity is a field that reproduces itself after undergoing a roundtrip inside the FP cavity [4]. The cavity modes of an FP etalon are composed of plane waves making a uniform angle to the optical axis [5,6]. As collimated beams approximate plane waves, they match a cavity mode when incident upon an FP etalon and are thus typically used to illuminate FP etalons.

However, collimated beams cannot be used if localized measurements of the FP etalon are required. Example applications include FP etalons used for spatially resolved ultrasound sensing [7], FP etalons fabricated on the tip of a single-mode fiber for miniaturized sensing [8], and Micro-ElectroMechanical System (MEMS)-based pixel arrays of FP etalons [9]. In these applications, focused Gaussian beam illumination is typically used, but the focused beam limits the visibility, FWHM, and sensitivity of the ITF [10]. The ITF is degraded under these circumstances because focused Gaussian beams, required to achieve a small illumination element size, do not match a FP cavity mode since Gaussian beams are composed of plane waves propagating with a spectrum of directions of relative the optical axis [11]. One solution to this problem is to use alternative FP cavity geometries, thus changing the cavity modes. For example, FP cavities containing waveguides [12], a concave mirror [13], and a metasurface [14] have all been demonstrated. In each of these cases, the FP cavity modes were modified to match a focused Gaussian beam. However, each of these approaches leads to additional manufacturing challenges, and so designing a beam with a small illumination element size that is matched to the modes of a standard FP etalon is preferable.

Bessel beams are a type of beam which possess these attributes. Bessel beams with core diameters of tens of µm are formed by interfering azimuthally symmetric plane waves propagating with a single polar direction relative to the optical axis [15]. Thus, Bessel beam illumination could achieve localized measurements of FP etalons while matching an FP cavity mode as required to measure ITFs with high visibility, narrow FWHM, and high sensitivity. To test this hypothesis, in this Letter, we study the feasibility of using Bessel beams to illuminate FP etalons.

## Methods.

**Fig. 1.**
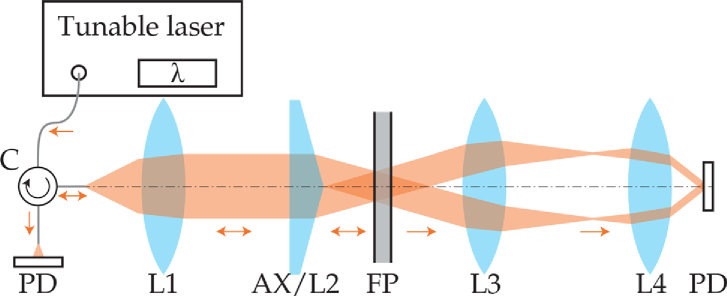
Diagram of the system used to measure ITFs using Bessel beam illumination. To achieve Gaussian beam illumination, the axicon was replaced by a lens. The abbreviations stand for circulator (C), photodiode (PD), lens (L), and axicon (A).

We investigated the feasibility of using Bessel beam illumination by comparing ITFs measured using Gaussian and Bessel beam illumination. In particular, for each beam type, a nearly collimated beam and a focused beam were used to allow comparison between ITFs for different core sizes. The optical system depicted in Fig. 1 was designed to simultaneously acquire ITFs for light reflected and transmitted by the FP etalon. In this system, Bessel beam illumination was replaced by Gaussian beam illumination by replacing the axicon in Fig. 1 with a lens.

Light from a continuous tunable laser (TSL-570, Santec) was coupled into a single-mode circulator (6015-3-APC, Thorlabs) with Mode Field Diameter (MFD) of (10.4±0.5) µm. Light exiting the circulator was collimated by a fixed length collimator (F220APC-1550, Thorlabs) and focused onto the FP etalon using an axicon or a lens. Light reflected by the FP etalon then propagated back through the collimator and was coupled back into the circulator, which redirected the light to an Indium Gallium Arsenide (InGaAs) photodiode (G9801-22, Hamamatsu). Light transmitted through the FP etalon propagated through a 4f system (LA1509-C-ML and LA1027, Thorlabs) with magnification of 0.35 to ensure that the full extent of the beam was detected by a second InGaAs photodiode (SM05PD5A, Thorlabs). The signals from both photodiodes were amplified using transimpedance amplifiers and measured with an oscilloscope (PicoScope 6403D, Pico Technology).

**Fig. 2.**
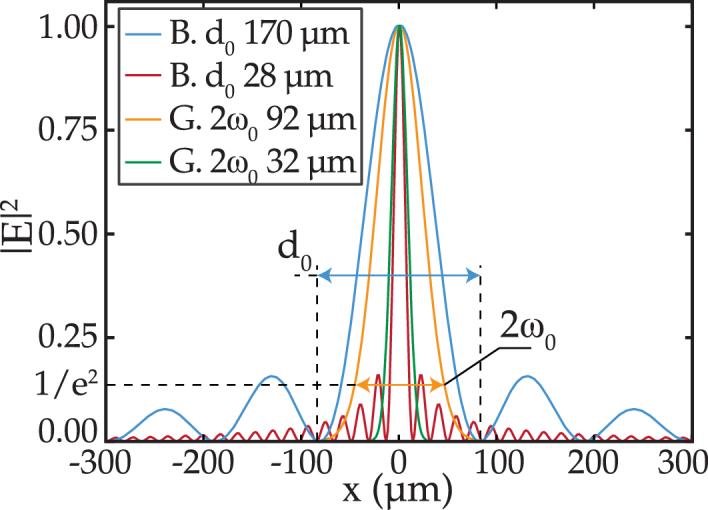
Simulated intensity cross sections across the center of the beams. The abbreviations stand for (G) Gaussian and (B) Bessel.

The nearly collimated Bessel beam was generated using an axicon (AX251-C, Thorlabs) yielding a core size, $d_{0}$ , defined as the diameter of the innermost dark fringe, of 170 µm and a depth of focus of 31 cm [15]. Another axicon (AX255-C, Thorlabs) was used to generate the second Bessel beam with a core size of 28 µm and a depth of focus of 5 cm. Similarly, lenses were used in place of the axicons to focus two Gaussian beams onto the FP. The Gaussian beams had waists, $2\omega_{0}$ , defined as the full width at 1/e^2^ of the intensity profile, of 92 µm and 32 µm, respectively. The Gaussian beams were formed by using the lenses LA1805-CL and LA1131-C-ML (Thorlabs) as L2. The radial profiles of the beams were computed and are shown in Fig. 2.

The FP etalon used had a 102 µm thick cavity made of fused silica surrounded by two dielectric mirrors [10]. Each mirror was made of a stack of alternating dielectric layers of Zinc Sulfide (ZnS) and Sodium Hexafluoroaluminate (Na_3_AlF_6_), each with a design optical thickness of a quarter of the reference wavelength of 1402 nm. The mirrors had a reflectivity varying from 97.2 % to 99.2 % in the wavelength range of the tunable laser (1500 nm to 1630 nm).

Simulated ITFs were computed to compare with the experimental measurements. The optical fields were propagated through the entire optical system by simulating the optical components making up the system sequentially, in the order in which the components appear in the light path of the optical system [16]. The optical components were simulated as (i) light exiting and coupling into the single-mode fibre, assuming the fibre mode to be Gaussian [17]; (ii) a phase-screen model for the axicon [18]; (iii) lenses using Fourier optics [19]; and (iv) an FP etalon using a dielectric stack model [20] in which the FP etalon was described by the refractive indices and thicknesses of all its layers according to the manufactured specifications [10]. The characteristics of all optical components (such as the focal length) were considered as specified by the manufacturers. The simulations were performed using Jolab [21], and the open-source code is available in Ref. [22].

## Results.

**Fig. 3.**
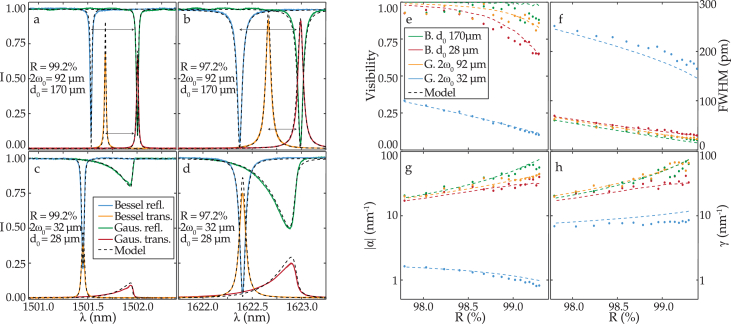
(a)–(d) ITFs acquired using different Bessel and Gaussian illumination beams and different mirror reflectivities (R). The modelled fringes were shifted along the wavelength axis (≈0.5 nm) to overlap with the experimental data for comparison. The fringes for Bessel beam interrogation in (a), (b) were shifted according to the arrow for display purposes. (e)–(h) Visibility, FWHM, minimum ( γ ), and maximum ( α ) ITF derivative with respect to wavelength as a function of mirror reflectivity ( R ). The metrics were computed from the ITFs measured in reflection.

A total of 16 ITFs were measured, for each Bessel and Gaussian beam and for each mirror reflectivity. A representative set of ITFs is plotted in Figs. 3(a)–3(d). As is visible in Fig. 3, when using a Gaussian beam with a 92 µm waist, the ITF had a relatively high visibility and low FWHM. However, upon reducing the beam waist to 32 µm, the visibility reduced, the FWHM increased, and the ITF became asymmetric [23]. In comparison, when using a 170 µm wide Bessel beam, the ITF also had high visibility and low FWHM, as for the 92 µm waist Gaussian beam. When reducing the Bessel core size to 28 µm, the ITF remained symmetric, the visibility reduced, and the FWHM increased. However, both of these changes were less significant than in the Gaussian case. To better visualize the trends, various metrics were computed and plotted as a function of mirror reflectivity. The metrics considered were visibility, FWHM, minimum ( γ ), and maximum ( α ) ITF derivative with respect to wavelength (which is proportional to the FP etalon sensitivity). These metrics were defined as

(1) $$\text{Visibility}=\left\{ \begin{array}{c} \frac{\max(\text{ITF})-\min(\text{ITF})}{\max(\text{ITF})+\min(\text{ITF})},\;\text{in reflection}\\ \max(\text{ITF}),\;\text{in transmission} \end{array}\right.$$

(2) $$\alpha =\max\left(\frac{d\;\text{ITF}}{d\;\lambda }\right),$$

(3) $$\gamma =\min\left(\frac{d\;\text{ITF}}{d\;\lambda }\right).$$

The metrics for ITFs measured in reflection are plotted in Fig. 3(e)–3(h). As visible in Fig. 3(e)–3(h), when reducing the Gaussian beam waist from 92 µm to 32 µm, all metrics were impacted. In particular, the visibility reduced by a factor of 9, the FWHM increased by a factor of 5, the minimum and maximum ITF derivatives reduced by a factor of 10 and 100, respectively. When reducing the Bessel core from 170 µm to 28 µm, the metrics changed considerably less than for the Gaussian case. In particular, the visibility reduced by a factor of 1.3, the FWHM increased by a factor of 1.4, and the minimum and maximum ITF derivatives reduced by a factor of 1.1 and 1.2, respectively. Thus, when using focused beam illumination, Bessel beams with a given core size provided higher visibility, lower FWHM, and higher sensitivity ITFs than for a Gaussian beam with similar beam waist.

As visible in Fig. 3, both modelled and experimented ITFs were in agreement, thus validating the model to simulate FP etalons illuminated with both Gaussian [10] and Bessel beams. The mismatch between experimental and modelled data was due to imperfections in the fabrication process of the mirrors. This is because the model simulated the mirrors according to their specification and did not account for imperfections due to the manufacturing tolerances [10].

## Discussion.

**Fig. 4.**
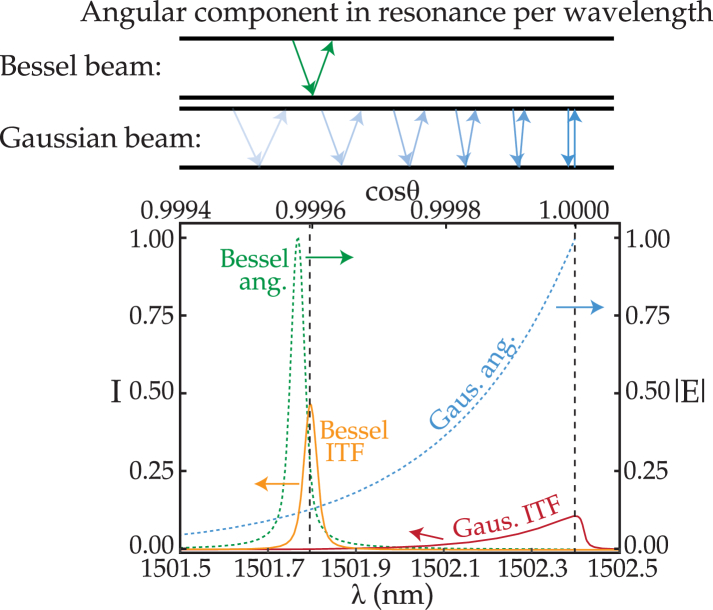
Angular components in resonance per wavelength along the ITF for a Bessel and a Gaussian beam. The directions of propagation sketched in the top part of the figure are for illustrative purposes only. The angular components (Bessel ang. and Gaus. ang.) are only plotted relative to the cos⁡θ axis. The ITFs are plotted against both the cos⁡θ and wavelength axes. The data was computed assuming a Bessel beam with $d_{0}$ of 28 µm and a Gaussian beam with a beam waist $2\omega_{0}$ of 32 µm. The Bessel ITF and angular spectrum peak do not align perfectly due to small changes in the light phase upon reflection by the mirrors [5]

We studied the structure of Bessel beams to understand why ITFs resulting from Bessel beam illumination possess superior characteristics. The core of a Bessel beam is formed by interfering waves propagating with the same polar direction relative to the optical axis. Thus, the angular spectrum of the Bessel beam, i.e., the beam decomposition into plane wave components propagating with different directions of propagation [11], has a narrow angular distribution, as visible on the angular spectrum plotted in Fig. 4. In contrast, a focused Gaussian spot is formed by interfering waves propagating with a wider angular distribution according to the numerical aperture of the optical system [11], as also shown in Fig. 4.

An FP etalon cavity has a continuous distribution of modes [5]. In particular, any beam propagating with a single polar direction of propagation is a mode of an FP etalon, and the wavelength at which the beam is in resonance is given by [1]:

(4) 2nhcos⁡θ=Zλ,

where n is the real part of the cavity refractive index, h is the cavity thickness, θ is the polar direction of propagation, and Z is an arbitrary integer. Thus, only a portion of Gaussian beam’s angular components is in resonance, at a given wavelength, due to its wide angular distribution. Therefore, a Gaussian beam has a spectrum of wavelengths at which resonance occurs [5], as shown in the ITF plotted in Fig. 3. Consequently, the ITF becomes asymmetric, the visibility reduces, and the FWHM increases. For a Bessel beam, as the beam propagates with a single polar direction, all the angular components forming the beam can be in resonance at a single resonance wavelength. Therefore, the ITF has a higher visibility and a lower FWHM relative to Gaussian beam illumination.

ITFs obtained using Bessel beams possess characteristics superior to those of Gaussian beams. However, a Bessel beam has sidelobes around the central core, as is visible in Fig. 2. The sidelobes increase the illumination element size within the FP etalon and, consequently, may impact the performance of the system for applications such as spatially resolved or miniaturized sensing. The proportion of beam energy in the sidelobes increases with the narrowing of the angular distribution of the Bessel beam. Therefore, since the ITF improves as the angular distribution narrows, the design of the Bessel beam must make a compromise between the proportion of beam energy in the sidelobes (which increases the illumination element size) and the ITF characteristics. The optical model, developed and experimentally validated in this Letter, can efficiently predict the evolution of the ITF as different Bessel beam designs are considered. Thus, the model could find application in optimizing the trade-off between the illumination element size and the ITF characteristics.

## Conclusion.

The feasibility of using Bessel beams to acquire ITFs with high visibility, narrow FWHM, and high sensitivity using FP etalons along with a small illumination element size was performed. Focused Bessel beams resulted in ITFs with higher visibility, narrower FWHM, and higher sensitivity when compared with focused Gaussian beams with a beam waist matching the Bessel core diameter. The improvement in the ITF was due to the Bessel beam matching the modes of the FP cavity, as Bessel beams propagate with a single polar direction. Thus, illuminating FP etalons with Bessel beams could, for example, provide a means to increase the sensitivity of spatially resolved FP sensors [7] or to increase the transmissivity of a pixel array of FP etalons [9].

## Data Availability

Data underlying the results presented in this paper are not publicly available at this time but may be obtained from the authors upon reasonable request.
